# Celecoxib prevents tumor necrosis factor-α (TNF-α)-induced cellular senescence in human chondrocytes

**DOI:** 10.1080/21655979.2021.2003661

**Published:** 2021-12-13

**Authors:** Qunli Wang, Qi Chen, Jie Sui, Yuanyuan Tu, Xiang Guo, Feng Li

**Affiliations:** a Department of Orthopaedic Center, Affiliated Haikou Hospital of Xiangya Medical College, Central South University, Haikou, Hainan, China; b Department of Orthopedics, The 928th Hospital of the Joint Logistic Support Force of the People’s Liberation Army, Haikou, Hainan, China; c Department of Orthopedics, The 904th Hospital of the Joint Logistic Support Force of the People’s Liberation Army, Changzhou, Jiangsu, China

**Keywords:** Celecoxib, osteoarthritis, chondrocytes, cell senescence

## Abstract

Osteoarthritis (OA) is a cartilage degenerative disease commonly observed in the elderly population and significantly impacts the normal life of OA patients. It has been reported that the development of pathological cell senescence in chondrocytes is involved in the pathogenesis of OA. Celecoxib is a common non-steroidal anti-inflammatory drug, and it has been recently reported to exert therapeutic effects on OA. However, its underlying mechanism is still unclear. The present study intends to explore its mechanism and provide fundamental evidence for the application of Celecoxib in the treatment of clinical OA. Tumor necrosis factor-α (TNF-α) was utilized to establish an *in vitro* model of chondrocytes senescence. The elevated reactive oxygen species (ROS) generation, increased cell cycle arrest in G0/G1 phase, reduced telomerase activity, and upregulated senescence-associatedβ-galactosidase (SA-β-Gal) staining were all observed in TNF-α-treated chondrocytes, which were then dramatically reversed by 10 and 20 μM Celecoxib. In addition, the upregulated DNA damage biomarkers, p-ATM, and p-CHK2, observed in TNF-α-treated chondrocytes were significantly downregulated by 10 and 20 μM Celecoxib. Lastly, the expression level of p21 and p53 was greatly elevated in chondrocytes by stimulation with TNF-α which was then pronouncedly repressed by treatment with Celecoxib. Taken together, our data reveal that Celecoxib ameliorated TNF-α-induced cellular senescence in human chondrocytes.

## Introduction

Osteoarthritis (OA) is a degenerative disease characterized by joint pain, stiffness, dysfunction, and even disability [1], which is commonly observed in the elderly population. According to reports, OA affects 10% of males and 18% of females over the age of 60 globally [2,3]. Approximately 250 million patients globally have been diagnosed with OA and in China, the morbidity of OA in the elderly population over the age of 75 is about 80% [3]. It is predicted that by 2030, OA will be the single disease with the highest disability rate in the world, significantly impacting the normal life and health of human beings. In previous decades, various studies focused on the inflammatory responses and degradation of the extracellular matrix (ECM) [4,5]. Recently, cell senescence in chondrocytes was proven to be closely associated with the pathogenesis of OA. However, the underlying mechanism is still unclear. As the articular cartilage ages, multiple signaling pathways change and impact the homeostasis of ECM, ultimately inducing the disruption of the cartilage structure and aggravation of the cartilage biomechanical property [6]. In geriatric diseases, the pathological changes in lesions are accompanied by chronic inflammation [7]. It is widely reported that the inflammatory pathways can be activated to induce damage to articular cartilage by various types of OA inducers, such as impaired autophagy, loss of apoptotic body clearance, protein misfolding, and oxidative stress [8]. The release of inflammatory factors in the serum, such as interleukin (IL)-1, IL-6, and tumor necrosis factor-α (TNF-α), is proportionally elevated with age and accelerates the aging of cartilage tissues [9]. In healthy cartilage tissues, chondrocytes are in a resting state with stable functions [10]. Cell senescence is mainly characterized by growth stagnation, metabolic changes, and loss of proliferation, which is different from the resting state [11]. Physiological cell senescence develops when the telomerase is exhausted after multiple replications, while pathological cell senescence is induced by DNA damage caused by pressure or inflammation [10]. DNA damage-induced cell senescence can be reflected by the upregulated levels of proteins such as p-ATM and p-CHK2 [12,13]. Recently, it has been reported that cell senescence in chondrocytes plays a critical role in the progression of cartilage regeneration [14]. In mice, OA develops after injecting senescent chondrocytes into the knee joint cavity [15]. Furthermore, specifically removing p16 marked senescent chondrocytes, significantly alleviates the pathological symptom in OA mice [16]. Therefore, cell senescence in chondrocytes is a promising target for the treatment of OA.

Celecoxib is a common non-steroidal anti-inflammatory drug approved for the treatment of rheumatoid arthritis due to its anti-inflammatory property by the US Food and Drug Administration (FDA), it exerts anti-inflammatory effects by suppressing the production of prostaglandin through inhibiting the activity of cyclooxygenase-2 (COX-2) [17]. Recently, Celecoxib has been reported to show significant therapeutic effects in arthritis [18]. However, the underlying mechanism remains unclear. The present study aims to investigate whether Celecoxib possesses a beneficial effect against TNF-α-induced cellular senescence in C-28/I2 chondrocytes.

## Materials and methods

### Cell culture and treatments

Human C-28/I2 chondrocytes were obtained from Sigma-Aldrich (#SCC043) and cultured in Dulbecco’s modified eagle medium (DMEM)/F12 medium containing 10% fetal bovine serum (FBS) and 1% Penicillin/Streptomycin under 37°C and 5% CO_2_.

### Cell viability

To determine the optimized concentration of Celecoxib (#SC58635 Invivochem, Libertyville, USA) for incubating C-28/I2 cells, cells were treated with Celecoxib at the concentrations of 0, 1, 5, 10, 20, 100, 200 μM for 24 hours, followed by measuring the cell viability using the Cell Counting Kit-8 (CCK-8) assay. In brief, cells were seeded on a 96-well plate and incubated for 24 hours, followed by replacing the culturing medium supplemented with 10% CCK-8 (#HY-K0301 MedChemExpress, Shanghai, China) solution. Lastly, 2 hours later, the microplate reader (Molecular Devices, California, USA) was utilized to measure the absorbance at 450 nm wavelength with a reference wavelength of 570 nm.

### ROS determination with Dichlorodihydrofluorescein diacetate (DCFH-DA) staining

C-28/I2 chondrocytes were seeded on a 96-well plate. After necessary treatment, 10 μM DCFH-DA was added to serum-free DMEM/F12 medium. Then, cells were incubated for 30 min followed by being washed using the serum-free DMEM/F12 medium. Lastly, the microplate reader (Molecular Devices, California, USA) was used to measure the fluorescent intensity at 535 nm [19]. Fluorescent images were captured with a Nikon Eclipse T300 fluorescence microscope.

### Cell cycle assay

C-28/I2 chondrocytes were collected and fixed with 70% cold ethanol at −20°C for 12 hours, followed by being treated with 1 mg/mL RNase A and 10 µg/mL propidium iodide (PI) for 30 min in the dark at 37°C. Then, cells were loaded onto the FACS Canto-II analyzer (BD, California, USA) for cell cycle analysis [20].

### Telomerase activity

Telomerase activity was measured using the telomeric repeat amplification protocol (TRAP) and the TeloTAGGG Telomerase PCR ELISA Kit (#11,854,666,910, Sigma-Aldrich, USA). For the first step, 3 µL cell extract was mixed with 25 µL reaction mixture, 22 µL DNase-free water, and it was subjected to the following PCR protocols: primer elongation at 25°C for 30 min, telomerase inactivation at 94°C for 5 min, 30 cycles of denaturation at 94°C for 30 s, annealing at 50°C for 30 s, and polymerization at 72°C for 90 s, followed by 72°C for 10 min, and held at 4°C. The hybridization and ELISA procedure were performed as per the manufacturer’s instructions. OD value was recorded at 450 nm with a reference wavelength of 630 nm.

### Senescence-associated -β- galactosidase (SA-β-Gal) staining assay

After necessary treatment, cellular senescence was assayed using an SA-β-Gal commercial kit (#CBA-230, Cell Biolabs, USA). Cells were then washed with PBS and fixed with fixing solution for 5 min at room temperature. After three washes with PBS, cells were loaded with 2 mL of freshly prepared Cell Staining Working Solution and incubated at 37°C for 6 hours. Cells were then washed with PBS and the blue stained senescence cells were visualized using a light microscope.

### Western blot assay

The lysis buffer was utilized to extract total proteins from C-28/I2 chondrocytes and the isolated proteins were quantified with a BCA kit (#71,285-M, Sigma-Aldrich, USA), followed by loading 30 μg proteins of each sample onto the 12% SDS-PAGE. After resolving for 1 hour, proteins in the gel were further transferred onto the PVDF membrane (#1,620,256 Bio-Rad, USA), and incubated with 5% BSA. Then, the membrane was incubated with the primary antibodies against p-ATM (1:800, # sc-47,739 Santa Cruz Biotechnology, Texas, USA), ATM (1:3000, # sc-135,663 Santa Cruz Biotechnology, Texas, USA), p-CHK2 (1:800, #ab278548 Abcam, Cambridge, UK), CHK2 (1:2000, #sc-17,747 Santa Cruz Biotechnology, Texas, USA), p21 (1:1000, #sc-6246 Santa Cruz Biotechnology, Texas, USA), p53 (1:2000, #sc-126 Santa Cruz Biotechnology, Texas, USA), and β-actin (1:5000, #sc-47,778 Santa Cruz Biotechnology, Texas, USA). Then the secondary antibody (1:2000, #sc-2359 Santa Cruz Biotechnology, Texas, USA) was utilized to incubate with the membrane for 1.5 h under room temperature. Lastly, the bands were incubated with the ECL solution, followed by quantification using the Image J software [21].

### Statistical analysis

The data were analyzed using the GraphPad software and presented as mean ± SD. The t-test was used to compare two independent data sets and the data among groups were compared using the Analysis of Variance (ANOVA) method, while p < 0.05 was taken as a significant difference.

## Results

In the present study, the protective effects of Celecoxib on TNF-α-induced cellular senescence were assessed. Firstly, the cytotoxicity of Celecoxib was tested to choose optimal concentrations. Secondly, we found that Celecoxib could ameliorate oxidative stress and mitigate cellular senescence by increasing telomerase activity, preventing cell cycle arrest in the G0/G1 phase, and attenuating DNA damage. Importantly, we found that Celecoxib reduced the expression of p21 and p53.

### The cytotoxicity of Celecoxib in human C-28/I2 chondrocytes

**Figure 1.**
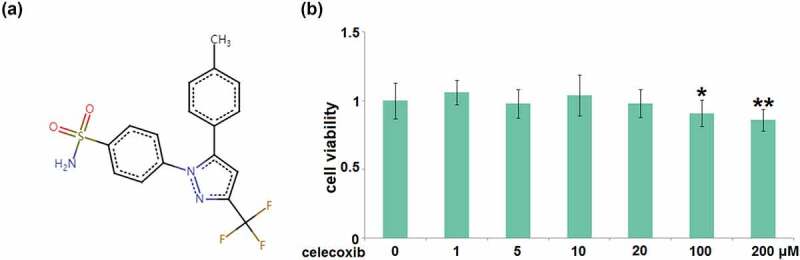
The cytotoxicity of celecoxib in human C-28/I2 chondrocytes. Cells were treated with celecoxib at concentrations of 0, 1, 5, 10, 20, 100, 200 μM for 24 hours. (a) The molecular structure of celecoxib; (b) The effects of celecoxib in cell viability of human chondrocytes (*, **, P < 0.05, 0.01 vs. vehicle group)

To determine the optimized concentration of Celecoxib to incubate C-28/I2 cells, cells were treated with Celecoxib at the concentrations of 0, 1, 5, 10, 20, 100, 200 μM for 24 hours, followed by measuring their viability using the CCK8 assay. The molecular structure of Celecoxib is shown in Figure 1a. Compared to the control, the cell viability (Figure 1b) was slightly changed as the concentration of Celecoxib increased from 1 to 20 μM. It was significantly decreased when the concentration of Celecoxib was higher than 20 μM, therefore, 10 and 20 μM were utilized as the incubation concentrations of Celecoxib in human C-28/I2 chondrocytes.

### The effect of Celecoxib on ROS induced by TNF-α in human C-28/I2 chondrocytes

**Figure 2.**
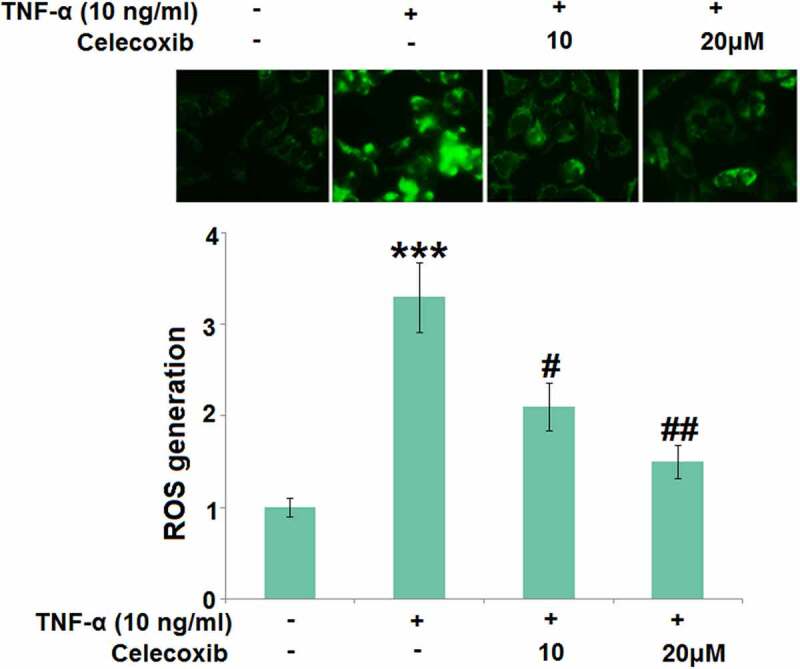
Celecoxib reduced ROS production in TNF-α-challenged human C-28/I2 chondrocytes. Cells were treated with TNF-α (10 ng/ml) in the absence or presence of Celecoxib at concentrations of 10 and 20 μM for 24 hours. ROS generation was labeled by green fluorescence (***, P < 0.005 vs. vehicle group; #, ##, P < 0.05, 0.01 vs. TNF-α group)

Chronic inflammation is the main inducer for the development of OA and oxidative stress is one of its main pathological characteristics. In the present study, 10 ng/mL TNF-α was utilized to induce the *in vitro* model in chondrocytes. C-28/I2 chondrocytes were treated with TNF-α (10 ng/ml) in the absence or presence of 10 and 20 μM Celecoxib for 24 hours. We found that the generation of ROS in chondrocytes was significantly elevated by stimulation with 10 ng/mL TNF-α, then greatly suppressed by 10 and 20 μM Celecoxib (Figure 2), indicating that the oxidative stress in TNF-α-treated chondrocytes was dramatically alleviated by Celecoxib.

### The effect of Celecoxib on the cell cycle arrest in the G0/G1 phase induced by TNF-α human C-28/I2 chondrocytes

**Figure 3.**
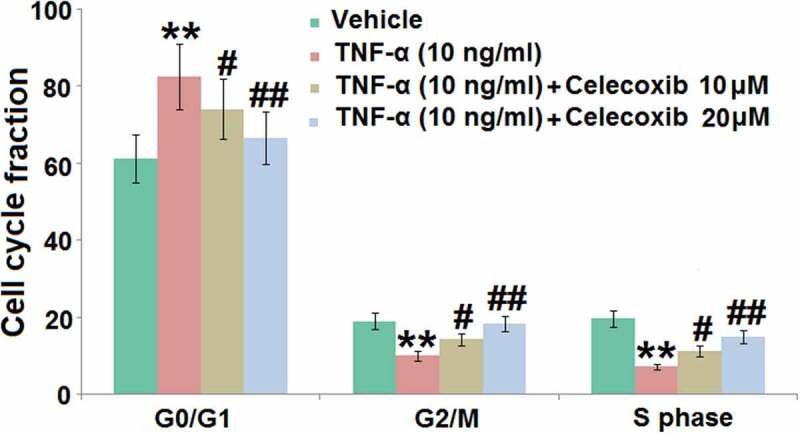
The effect of Celecoxib on the cell cycle arrest in the G0/G1 phase induced by TNF-α in human C-28/I2 chondrocytes. Cells were treated with TNF-α (10 ng/ml) in the absence or presence of Celecoxib at concentrations of 10, 20 μM for 7 days. Cell cycle fraction in the G0/G1 phase, G2/M phase, and S phase was calculated (***, P < 0.005 vs. vehicle group; #, ##, P < 0.05, 0.01 vs. TNF-α group)

DNA damage is the main inducer for pathological cell senescence, which results in the cycle arrest [22]. C-28/I2 chondrocytes were treated with 10 ng/mL TNF-α in the absence or presence of 10 and 20 μM Celecoxib for 7 days, followed by measuring the cell cycle using flow cytometry. In TNF-α-treated chondrocytes, significantly elevated cell fraction was observed in the G0/G1 phase, accompanied by the decline of cell fraction in the S and G2/M phase, both of which were dramatically reversed by the introduction of 10 and 20 μM Celecoxib (Figure 3), indicating that cell cycle arrest in TNF-α-treated chondrocytes was greatly ameliorated by Celecoxib.

### The effect of Celecoxib on the telomerase activity in TNF-α-stimulated human C-28/I2 chondrocytes

**Figure 4.**
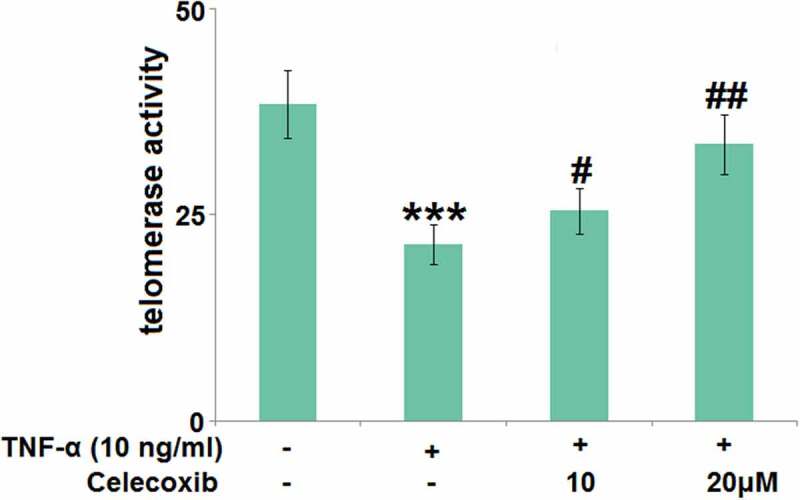
The effect of Celecoxib on the telomerase activity in TNF-α-challenged human C-28/I2 chondrocytes. Cells were treated with TNF-α (10 ng/ml) in the absence or presence of Celecoxib at concentrations of 10, 20 μM for 7 days. The telomerase activity was detected using a chemical kit (***, P < 0.005 vs. vehicle group; #, ##, P < 0.05, 0.01 vs. TNF-α group)

The declined activity of telomerase is the molecular marker for cell senescence [23]. We further measured the telomerase activity (Figure 4) using a chemical kit. After stimulation with TNF-α (#H8916 Sigma-Aldrich, Shanghai, China), the telomerase activity was significantly decreased from 38.5 to 21.4 IU/L, which was then greatly increased to 25.5 and 33.6 IU/L by 10 and 20 μM Celecoxib, respectively, indicating that Celecoxib protected the telomerase activity in TNF-α-stimulated human C-28/I2 chondrocytes.

### Celecoxib ameliorated cellular senescence against TNF-α in human C-28/I2 chondrocytes

**Figure 5.**
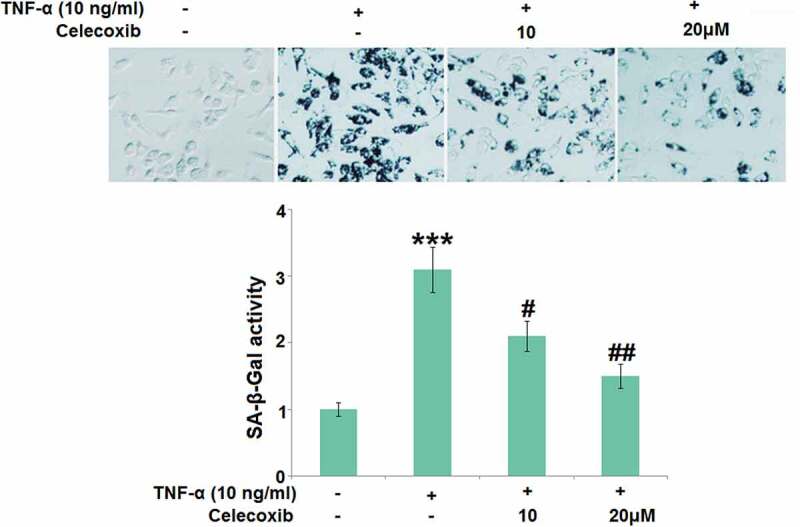
Celecoxib ameliorated cellular senescence against TNF-α in human C-28/I2 chondrocytes. Cells were treated with TNF-α (10 ng/ml) in the absence or presence of Celecoxib at concentrations of 10, 20 μM for 7 days. The SA-β-Gal activity was determined (***, P < 0.005 vs. vehicle group; #, ##, P < 0.05, 0.01 vs. TNF-α group)

Subsequently, SA-β-Gal activity in TNF-α-treated chondrocytes was further determined. We found that the SA-β-Gal activity (Figure 5) in C-28/I2 chondrocytes was significantly promoted by stimulation with TNF-α, then greatly repressed by 10 and 20 μM Celecoxib.

### The effect of Celecoxib on the activation of p-ATM and p-CHK2 induced by TNF-α in human chondrocytes

**Figure 6.**
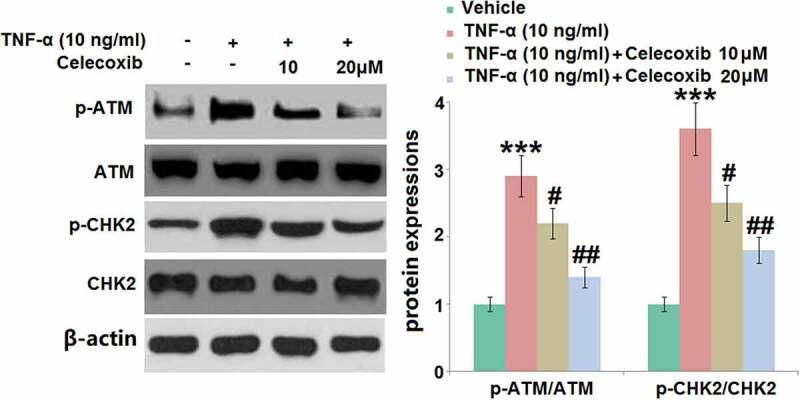
The effect of Celecoxib on the activation of p-ATM and p-CHK2 induced by TNF-α in human chondrocytes. Cells were treated with TNF-α (10 ng/ml) in the absence or presence of Celecoxib at the concentrations of 10, 20 μM for 6 hours. The expressions of p-ATM/ATM and p-CHK2/CHK2 were detected using western blots (***, P < 0.005 vs. vehicle group; #, ##, P < 0.05, 0.01 vs. TNF-α group)

We further investigated the biomarkers (p-ATM and p-CHK2) of DNA damage during the progression of pathological senescence [12]. The results of the Western blotting assay (Figure 6) show that the relative expression levels of p-ATM/ATM and p-CHK2/CHK2 were significantly elevated in TNF-α-treated chondrocytes, then greatly inhibited by 10 and 20 μM Celecoxib, indicating a promising protective effect of Celecoxib against TNF-α- induced DNA damage in human C-28/I2 chondrocytes.

### The effect of Celecoxib on the increased expression of p21 and p53 induced by TNF-α in human C-28/I2 chondrocytes

**Figure 7.**
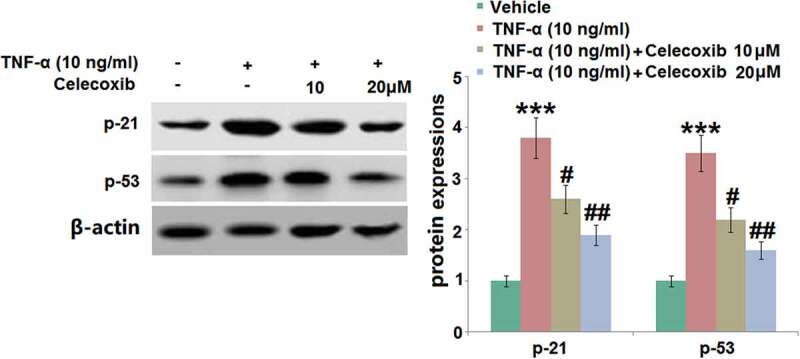
The effect of Celecoxib on the increased expression of p21 and p53induced by TNF-α in human C-28/I2 chondrocytes. Cells were treated with TNF-α (10 ng/ml) in the absence or presence of Celecoxib at concentrations of 10, 20 μM for 6 hours. The expressions of p21 and p53 were detected using western blots (***, P < 0.005 vs. vehicle group; #, ##, P < 0.05, 0.01 vs. TNF-α group)

P21 and p53 pathways are classic signaling pathways involved in the development of cell senescence [24]. As illustrated in Figure 7, p21 and p53 were found to be significantly upregulated in human C-28/I2 chondrocytes following TNF-α stimulation. They were then dramatically downregulated by 10 and 20 μM Celecoxib, indicating that the senescence-related pathways in TNF-α-treated human C-28/I2 chondrocytes were significantly repressed by Celecoxib.

## Discussion

Cell senescence is regularly triggered by several complicated elements, such as telomere shortening, genomic damage, and oxidative stress [25]. As normal cell replication develops, a recession is observed on the ability of cell proliferation, differentiation, and physiology in chondrocytes. However, under consecutive external mechanical pressure, intrinsic hypoxia, and low pH, pathological cell senescence develops in chondrocytes [26]. SA-β-Gal is the most reliable biomarker identified for cell senescence [27]. Price [28] reported that the rare SA-β-Gal was found to be expressed in cartilage tissues isolated from healthy subjects but it was significantly upregulated in cartilage tissues of OA patients. Gao proved that there is a correlation between the expression level of SA-β-Gal and the severity of OA [29]. In the present study, under the stimulation with TNF-α, significantly higher activity of SA-β-Gal was observed, indicating that cell senescence in chondrocytes was induced by TNF-α, which was consistent with the results reported previously [30]. Telomere shortening is another important characteristic for cell senescence [31]. It is reported that pathological changes in OA progression can be aggravated by chondrocytes senescence induced by telomere shortening, which is triggered by the elevated telomerase activity [32]. We found that the telomerase activity in chondrocytes was significantly enhanced by stimulation with TNF-α. After treatment with Celecoxib, cell senescence in chondrocytes was dramatically repressed, which was identified by the decreased activity of telomerase and SA-β-Gal.

Chondrocytes are located in the hypoxia environment. However, under the pathological state, excessive ROS are produced by chondrocytes [33]. ROS suppress the synthesis and accumulation of proteoglycan and type II collagen in chondrocytes by activating the mitogen-activated protein kinase, further accelerating the degradation of cartilage tissues by disrupting the extracellular matrix. In addition, the pathological state of OA can be aggravated by the stimulation of ROS by upregulating the expression level of inflammatory factors, such as matrix metalloproteinase, inducible nitric oxide synthase, IL-6, IL-1β, and TNF-α [34]. We found that the ROS level in chondrocytes was significantly elevated by stimulation with TNF-α, indicating the activation of oxidative stress. After the introduction of Celecoxib, the state of oxidative stress was dramatically reversed. Series pathological changes are induced by ROS, including DNA damage, telomere shortening, chondrocytes senescence, degradation of ECM, and the disruption of cartilage tissues [35]. We found biomarkers of DNA damage, p-ATM, and p-CHK2, were dramatically upregulated in chondrocytes by stimulation with TNF-α, accompanied by the cell cycle arrest in the G0/G1 phase. DNA damage and cell cycle arrest were significantly reversed by Celecoxib, which further verified the protective effect of Celecoxib against TNF-α treated chondrocytes.

P21 is a marker protein for cell senescence, which is mainly regulated by the transcriptional activation function of p53 [36]. After binding with cyclin-dependent kinase (CDK), a complex of p21-cyclin-CDK-PCNA is formed by p21 to inactivate CDK and proliferating cell nuclear antigen (PCNA), preventing cells from entering the S phase [37]. The transcription of p21 can be induced by wild-type p53, which plays an important role in cell cycle arrest induced by DNA damage [38]. Therefore, p53/p21 is a critical axis involved in the development of cell senescence. In the present study, the p53/p21 pathway was significantly activated in TNF-α-treated chondrocytes, verifying the cell senescence in chondrocytes induced by TNF-α. After treatment with Celecoxib, the p53/p21 pathway was significantly suppressed, indicating that the protective effect of Celecoxib might be associated with the inactivation of p21 or p53. In future work, the regulatory effect of Celecoxib on the p53/p21 pathway and the involvement of the p53/p21 pathway in the protective effects of Celecoxib against OA will be further investigated by introducing a specific inhibitor of p53 or knocking down the expression of p53 in chondrocytes.

## Conclusion

In summary, our data reveal that Celecoxib ameliorated TNF-α-induced cellular senescence in human chondrocytes, exploring a novel mechanism whereby Celecoxib exerted its protective effects in osteoarthritis (OA).

## Data Availability

Requests for data and materials should be addressed to the corresponding author.
